# Nucleated Red Blood Cells: Could They Be Indicator Markers of Illness Severity for Neonatal Intensive Care Unit Patients?

**DOI:** 10.3390/children7110197

**Published:** 2020-10-27

**Authors:** Rozeta Sokou, Georgios Ioakeimidis, Maria Lampridou, Abraham Pouliakis, Andreas G. Tsantes, Argyrios E. Tsantes, Nicoletta Iacovidou, Aikaterini Konstantinidi

**Affiliations:** 1 Neonatal Intensive Care Unit, Nikaia General Hospital “Aghios Panteleimon”, 184 54 Piraeus, Greece; sokourozeta@yahoo.gr (R.S.); giorgos.ioakeimidis@gmail.com (G.I.); litsamaria@hotmail.com (M.L.); 2 2nd Department of Pathology, School of Medicine, Attikon Hospital, National and Kapodistrian University of Athens, 124 62 Athens, Greece; apou1967@gmail.com; 3 Laboratory of Haematology and Blood Bank Unit, Attikon Hospital, School of Medicine, National and Kapodistrian University of Athens, 124 62 Athens, Greece; andreas.tsantes@yahoo.com (A.G.T.); atsantes@yahoo.com (A.E.T.); 4 Neonatal Department, School of Medicine, Aretaieion Hospital, National and Kapodistrian University of Athens, 124 62 Athens, Greece; niciac58@gmail.com

**Keywords:** critically ill neonates, nucleated red blood cells, mortality, morbidity, perinatal hypoxia, sepsis

## Abstract

Background: We aimed to assess whether nucleated red blood cells (NRBCs) count could serve as a diagnostic and prognostic biomarker for morbidity and mortality in critically ill neonates. Methods: The association between NRBCs count and neonatal morbidity and mortality was evaluated in an observational cohort of critically ill neonates hospitalized in our neonatal intensive care unit over a period of 69 months. The discriminative ability of NRBCs count as diagnostic and prognostic biomarkers was evaluated by performing the Receiver Operating Characteristics (ROC) curve analysis. Results: Among 467 critically ill neonates included in the study, 45 (9.6%) of them experienced in-hospital mortality. No statistically significant difference was found with regards to NRBCs count between survivors and non-survivors, although the median value for NRBCs was sometimes higher for non-survivors. ROC curve analysis showed that NRBCs is a good discriminator marker for the diagnosis of perinatal hypoxia in neonates with area under the curve (AUC) [AUC 0.710; 95% confidence interval (CI), 0.660–0.759] and predominantly in preterm neonates (AUC 0.921 (95% CI, 0.0849–0.0993)) by using a cut-off value of ≥11.2%, with 80% sensitivity and 88.7% specificity. NRBCs also revealed significant prognostic power for mortality in septic neonates (AUC 0.760 (95% CI, 0.631–0.888)) and especially in preterms with sepsis (AUC 0.816 (95% CI, 0.681–0.951)), with cut-off value ≥ 1%, resulting in 81.6% sensitivity and 78.1% specificity. Conclusion: NRBCs count may be included among the early diagnostic and prognostic markers for sick neonates.

## 1. Introduction

Critically ill neonates constitute a fragile population with very special characteristics compared to children and adults; hence, early diagnosis and management of critical illness are of great importance for their short-term outcome and lifelong prognosis. Various scoring systems have been established to estimate illness severity in neonates, such as the Score for Neonatal Acute Physiology (SNAP), SNAP-Perinatal Extension (SNAPPE) and SNAPPE-II, Neonatal Multiple Organ Dysfunction (NEOMOD) score, and Clinical Risk Index for Babies scoring system (CRIB II) [1,2,3,4]. These systems aim to achieve early identification of ill neonates with increased risk of morbidity and mortality and may contribute to improved patient care.

Advances in neonatal medicine have focused on several clinical and laboratory parameters as diagnostic and prognostic markers. The management of critically ill newborns such as septic newborns and those with perinatal hypoxia might be improved and more targeted using novel biomarkers, which could help for the detection of the disease within the critical time window. Previous studies have reported that hematological and serum biochemical variables such as leukocyte and neutrophil count, nucleated red blood cells (NRBCs), C-reactive protein (CRP), procalcitonin (PCT), arterial blood gas analysis, lactate, hepatic enzymes, plasma creatinine (Cr), and troponin high sensitivity (hsTn) have been used for this purpose [5,6,7,8,9,10,11].

NRBCs are early erythrocyte precursors not detectable in the peripheral blood of healthy adults under normal conditions. Contrarily, NRBCs constitute a normal finding in the circulation of the fetus and the neonates during the first week of life, depending on their gestational age and health status [12]. NRBCs count reflects high production of erythropoietin as a result of decreased arterial oxygen partial tension and/or increased concentrations of inflammatory cytokines. In critically ill adults, NRBCs are strongly prognostic of mortality [13]. In a recent study, Menk et al. [14] reported that the presence of NRBCs in the circulation might be regarded as a marker of disease severity indicating a higher risk of intensive care unit (ICU) death. Although, several studies have reported that elevated NRBCs count correlates with perinatal hypoxia and inflammation severity (sepsis) [6,7,15,16,17]. Christensen et al. [18] found that “neonates with an elevated NRBC count at birth had the onset of hypoxia at least 28 to 29 h before birth”. To date, relevant data regarding NRBCs diagnostic and prognostic value in critically ill neonates are limited.

Our aim was to investigate if the detection of elevated NRBCs count in critically ill neonates could serve as diagnostic and prognostic marker of neonatal morbidity and mortality.

## 2. Material and Methods

### 2.1. Study Design

This single-center, observational study was conducted at the Neonatal Intensive Care Unit (NICU) of Nikaia General Hospital “Aghios Panteleimon”, Piraeus, Greece, from July 2014 to April 2020. The study protocol, designed, conducted, and reported in compliance with the Declaration of Helsinki, was approved by the Institutional Review Board of Nikaia General Hospital (15 July 2014, 32/2). Parental informed consent was obtained prior to recruitment.

### 2.2. Participants

**Figure 1:**
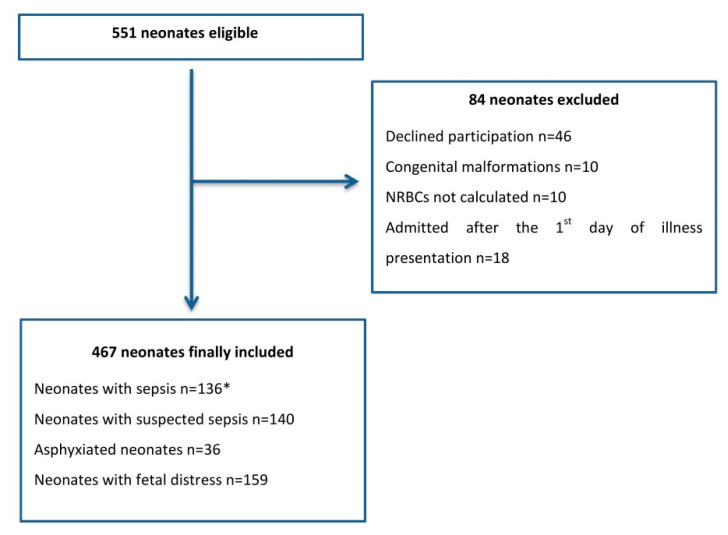
Flowchart of study population. * 4 neonates with perinatal hypoxia were also included in this subgroup, as they suffered from late-onset sepsis. Abbreviation: NRBCs, nucleated red blood cell counts.

Patients eligible for data collection were critically ill neonates hospitalized in our NICU during the study period. Critically ill neonates were characterized as: preterm neonates with respiratory distress syndrome (RDS), neonates with suspected or confirmed sepsis, and neonates with perinatal asphyxia/fetal distress. The definitions and the inclusion criteria for neonates mentioned above are in accordance with References [19,20,21]. Neonates with congenital malformations were excluded from the study. Data for the recruitment procedure are presented in the flow chart in Figure 1.

### 2.3. Variables’ Measurements

Data on demographics (birth weight, gestational age, sex), maternal and pregnancy history, neonatal physiological parameters, clinical findings, laboratory results, day of establishing full enteral feeding, and length of hospital stay were recorded for all study neonates. For preterm neonates (gestational age <37 weeks), severe morbidity such as respiratory distress syndrome (RDS) [19], neonatal sepsis [22], bronchopulmonary dysplasia (BPD) [23], necrotizing enterocolitis (NEC) [24], retinopathy of prematurity (ROP) [25], intraventricular hemorrhage (IVH) ≥ grade 2 [26], periventricular leukomalacia (PVL) [27], and patent ductus arteriosus (PDA) [28], were recorded. Morbidities recorded for term neonates were: respiratory morbidity including supplemental oxygen therapy, need for continuous positive airway pressure (CPAP) or ventilation support [29], sepsis, asphyxia [30], hypoxic ischemic encephalopathy (HIE) [31], and seizures. Mortality was defined as death before discharge. Delivery room deaths were not included as data were not available. Multiple organ dysfunction (MOD) was defined as the presence of dysfunction of at least two organ systems [32]. Organ involvement was determined by using laboratory tests and clinical evaluation.

In all study neonates, the SNAPPE score and modified NEOMOD scoring system [33] were calculated on admission to the NICU (for preterm neonates with RDS or neonates with perinatal asphyxia/fetal distress) and on the onset of the disease (for neonates with suspected or confirmed sepsis).

In the first 6–12 h of life (for preterm neonates with RDS and those with perinatal asphyxia/fetal distress), prior to initiating antibiotic therapy—if required, blood specimens for culture and biochemical tests (serum glutamil oxaloacetic transaminase (SGOT), complete blood count, (CRP) were performed, while on the second day of life (DOL), further biochemical parameters (sodium, potassium, SGOT, serum glutamic pyruvic transaminase (SGPT), blood urea nitrogen (BUN), creatinine plasma level (Cr), bilirubin, albumin (ALB)) were measured in all neonates, as per our NICU protocol. As for neonates with suspected or confirmed sepsis, the aforementioned laboratory tests were performed and recorded at the onset of clinical deterioration [median day of life: 12, interquartile range (IQR) 7–20]. Chest radiograph was performed whenever clinically indicated.

Complete blood count was performed on a Sysmex XE-2100 analyzer (Roche, IL, USA). Peripheral blood smears prepared with Giemsa stain were examined, and band forms, NRBCs, myelocytes and metamyelocytes (evaluated as immature neutrophils in leukocyte formula), and Immature to Total Neutrophil Ratio (I/T ratio) were calculated.

NRBCs measurement, expressed as NRBC count per 100 White Blood Cells (WBC), was carried out both automatically by the formerly mentioned analyzer and also by manual correction so that the results would be accurate.

### 2.4. Statistical Analysis

Descriptive statistics of the baseline data are presented as mean ± standard deviation (SD), median (ranges), or as percentages when appropriate. The differences in categorized and continuous data were compared using the chi-square contingency test and the with Mann-Whitney U test. Time-to-event analysis was carried out in order to evaluate the variables independently associated with the in-hospital survival. Univariate Cox’s proportional hazards analysis for relevant prognostic variables was performed. The hazards ratios or relative hazards derived from Cox’s proportional hazards models are presented with 95% confidence intervals (CIs) and the respective *p*-values. A ratio higher than unity implies a higher probability of death than in the reference group.

NRBCs count was evaluated to predict mortality in the study group. Their discriminative ability as diagnostic and prognostic biomarkers was evaluated by performing the Receiver Operating Characteristics (ROC) curve analysis. The optimal cut-off thresholds were identified with the Youden index, and the sensitivity, specificity, positive and negative predictive values were also evaluated. All statistical tests were two-sided. Stata statistical software was used for all statistical analyses (Stata Corp., College Station, TX, USA). For all tests, a *p*-value lower than 0.05 indicates statistical significance.

## 3. Results

A total of 467 critically ill neonates were included in the study, of which 45 (9.6%) did not survive. The baseline characteristics of the neonates are presented in Table 1.

Comorbidities recorded in study neonates are presented in Table 2.

Subsequently, we investigated the role of several parameters, and specifically, NRBCs, in the survival of the neonates (Table 3).

No statistically significant difference between survivors and non-survivors regarding GA and BW was found. Almost all laboratory studied parameters had a significant role in the survival of the neonates except for neutrophils percentage, WBC, and NRBC. Although the median value for NRBCs was sometimes times higher for non-survivors, this finding did not allow for proving a statistical significance under the condition of *p* < 0.05, probably because of the great variability in NRBCs and the small number of cases. In addition, this analysis proved that all scoring systems (i.e., SNAPPE, modified NEOMOD, and TOLLNER scores) were significantly different between survivors and non-survivors.

The analysis of study population mortality (as a dependent variable) in relation to comorbidities revealed that all conditions had a significant role in survivability except for intrauterine growth retardation (IUGR) and NEC (Table 4).

The performance of ROC curve analysis for the discriminative ability of NRBCs as diagnostic and prognostic markers in all study neonates is displayed in Table 5.

**Figure 2:**
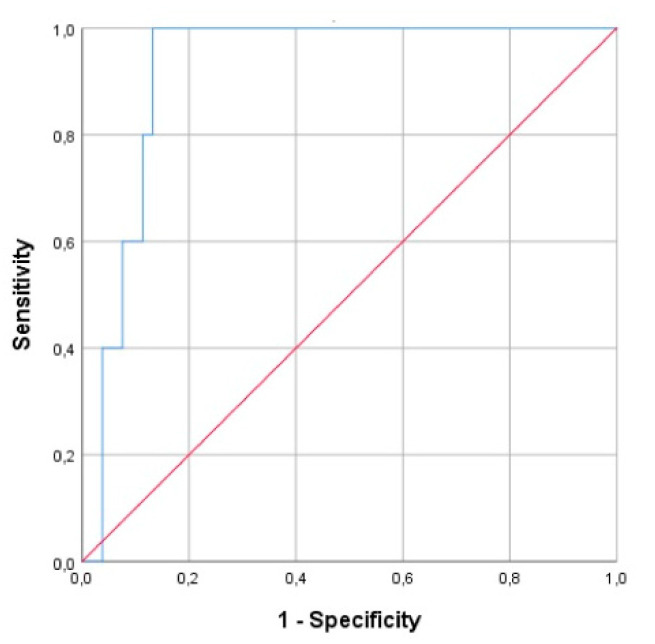
Area under the ROC curves of NRBCs for diagnosis of perinatal hypoxia in critically ill preterm neonates.

Subsequently, study neonates were subclassified as term and preterm neonates. ROC curve analysis showed that NRBCs is a good discriminator marker for the diagnosis of perinatal hypoxia in preterm neonates (Figure 2), with AUC 0.921(95% CI, 0.0849–0.0993). Using a cut-off value of ≥11.2%, NRBCs resulted in 80% sensitivity and 88.7% specificity.

**Figure 3:**
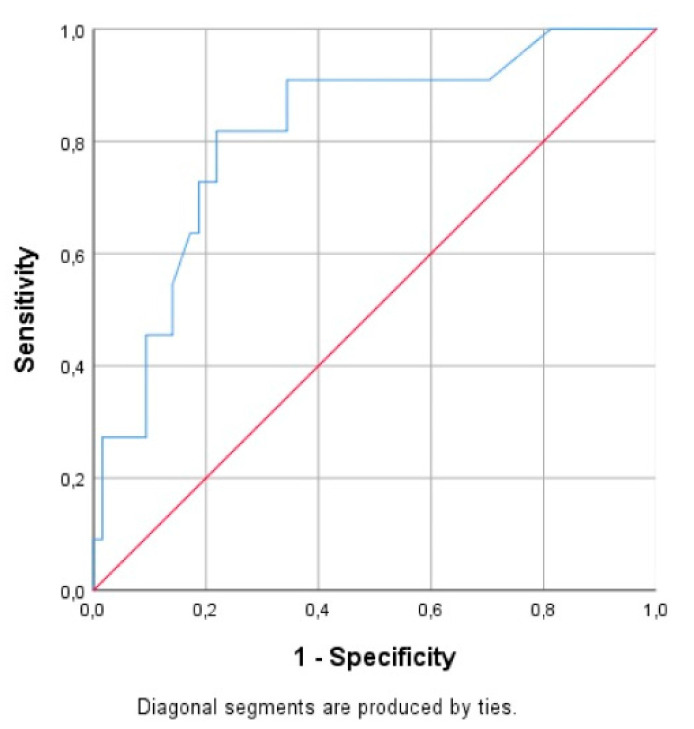
Area under the ROC curves of NRBCs for mortality prognosis in preterm neonates with sepsis.

NRBCs also revealed significant prognostic power for mortality in preterm neonates with sepsis (AUC 0.816 (95% CI, 0.681–0.951)), with cut-off value ≥1%, resulting in 81.6% sensitivity and 78.1% specificity (Figure 3). This low cut-off value probably attributed to the day of measurement (median: 12 (IQR 7–20)).

Contrarily, ROC curve analysis showed that NRBCs could not discriminate diagnosis or prognosis of sepsis or perinatal hypoxia between the term neonates of our study.

## 4. Discussion

In the present study, we evaluated the role of NRBCs count in the diagnosis and prognosis of morbidity and mortality in critically ill neonates. Our results revealed that NRBCs could serve, among others, as prognostic and diagnostic markers in this population, especially in preterm neonates. In healthy adults, NRBCs are not present in the peripheral blood but studies have reported their detection in 10–30% of critically ill patients [13]. Inflammation, hypoxia, or massive hemorrhage seem responsible for the appearance of NRBCs in peripheral blood, as these situations increase erythropoietic pressure and result in failure of the spleen to remove these cells from the circulation. Purtle et al. [34] demonstrated that the presence of NRBCs is associated with a significant increase in the odds of post-discharge hospital mortality in critically ill adults.

Schaer et al. [15] found that NRBCs are not an independent risk factor for bad outcomes in pediatric intensive care; however, they may have a prognostic value in the first month of life, although their association with outcome is much less pronounced beyond the neonatal period.

The presence of NRBCs in the circulation at birth and during the first week of life is an expected and rather physiological finding. In healthy neonates, NRBCs disappear from the peripheral blood within a few weeks of life, but they can return to the circulation in several pathological situations and their elevated value has been often correlated with poor short outcome and risk factors for poor neurodevelopment in neonates [16,35].

In septic neonates, data show that excess NRBCs in peripheral blood may help with the diagnosis of sepsis and correlates with the mortality of these neonates [36]. In our study, NRBCs showed a good performance as a prognostic marker for mortality in neonates with sepsis, especially in preterms.

NRBCs could also serve as hematologic markers for placental dysfunction, hypoxemia, and asphyxia as hypoxemia triggers erythropoietin release, resulting in stimulation of red blood cells. NRBCs count elevation at birth or persistence is linked to adverse short-term and long-term outcomes of neonates with perinatal hypoxia [18]. Boskabadi et al. [17] found that NRBC count in neonates with birth asphyxia were significantly higher than healthy controls and associated with poorer short-term outcome. In accordance with these findings, the current study showed that NRBCs count could serve as a discriminator marker for early diagnosis of perinatal hypoxia with excellent performance in preterm neonates (AUC 0.921) by using a cut-off value of ≥11.2%, with 80% sensitivity and 88.7% specificity. In a more recent study, Boskabadi et al. [7] demonstrated that theNRBC count can be used as a prognostic marker for neonatal asphyxia, which in combination with the HIE grade could indicate high infant mortality, and complications of asphyxia. Furthermore, NRBCs were found to be very useful in differentiating the neonates with hypoxic ischemic encephalopathy, leading to appropriate management and better outcome of these newborns [37]. The role of NRBCs count in critically ill neonates is confirmed by Morton et al. [38], who reported that among critically ill neonates, NRBCs are associated with significantly elevated mortality risk. In our study, although the median value for NRBCs count was found to be sometimes times higher for non-survivors, this parameter did not have good discriminative ability for predicting mortality in all the study population, probably because of the great variability in NRBCs and the small number of cases.

This single-center study also has some limitations which arise from the variability in NRBCs count and the small number of subjects. Another limitation might be the evaluation of NRBCs count only at the disease onset and this perhaps weakens the strength of the study with regards to the prognostic role of NRBCs in mortality.

In conclusion, NRBCs count may be encompassed among the early diagnostic and prognostic markers for neonatal intensive care unit patients. Further studies are needed to assess trends in NRBC values for critically ill neonates.

## Figures and Tables

**Table 1:** Baseline characteristics of the study population.

Descriptive Statistics	Value
Population (*n*)	467
Gender (Male)	300 (64.2%)
Gestational age (weeks)	35.3 ± 4.3
Birth weight (g)	2452.4 ± 976.9
Delivery mode (Caesarian session)	312 (66.8%)
Maternal diseases *	176 (42.1%)

* Preeclampsia, thyroid disease, gestational diabetes mellitus.

**Table 2:** Morbidities recorded in all neonates (*n* = 467).

	All Study Neonates *n* = 467	Term Neonates *n* = 248	Preterm Neonates *n* = 219	Terms vs. Preterms
				*p*-Value
Gestational age (weeks)	35.3 ± 4.3	38.7 ± 1.2	31.5 ± 3.2	0.000
Birth weight (g)	2452.4 ± 976.9	3147 ± 554	1665 ± 722	0.000
Death	45 (9.6%)	23 (9.3%)	22 (10%)	0.875
Respiratory Distress Syndrome	244 (52.6%)	88 (35.5%)	156 (71.2%)	0.000
Intrauterine Growth Retardation	56 (12.0%)	24 (9.7%)	32 (14.6%)	0.117
Sepsis	136 (29.1%)	52 (21%)	84 (38.4%)	0.000
Infection	140 (30.0%)	67 (27%)	73 (33.3%)	0.157
Perinatal hypoxia	195 (41.8%)	136 (54.8%)	59 (27%)	0.000
Perinatal asphyxia	36 (7.7%)	31 (12.5%)	5 (2.3%)	0.000
Fetal distress	159 (34%)	105 (42.3%)	54 (24.7%)	0.000
Acute Kidney Injury	75 (16.1%)	37 (14.9%)	38 (17.4%)	0.528
Disseminated Intravascular Coagulopathy	78 (16.7%)	36 (14.5%)	42 (19.2%)	0.174
Multiple Organ Dysfunction Syndrome	82 (17.6%)	45 (18.1%)	37 (16.9%)	0.808
**Bronchopulmonary Dysplasia**	70 (15%)	-	70 (32%)	-
Mild	19 (0.04%)		19 (8.7%)	
Moderate	14 (0.03%)		14 (6.4%)	
Severe	56 (12%)		56 (25.5%)	
**Retinopathy of Prematurity**	77 (16.5%)	-	77 (35.1%)	-
Laser treatment	29 (0.06%)		29 (13.2%)	
No treatment	48 (10.3%)		48 (22%)	
**Patent Ductus Arteriosus**	24 (5.1%)	-	24 (11%)	-
Pharmacological treatment	16 (0.03%)		16 (7.3%)	
Surgical ligation	5 (0.01%)		5 (2.3%)	
Conservative treatment	3 (0.006%)		3 (1.4%)	
**Periventricular Leukomalacia**	71 (15.2%)	-	71 (32.4%)	-
Intra-Ventricular Hemorrhage ≥ grade 2	61 (13%)	-	61 (27.8%)	-
Necrotizing Enterocolitis	15 (0.03%)	-	15 (6.8%)	-

**Table 3:** Baseline characteristics and laboratory data of survivors vs. non-survivors.

	Survivors (*n* = 422)	Non-Survivors (*n* = 45)	
Description	Median (IQR)	Median (IQR)	*p* Value
Gestational_Age (weeks)	37 (32–39)	37 (32–40)	0.1089
Birth_Weight (g)	2640 (1490–3230)	2550 (1420–3300)	0.9921
Base Deficit (mmol/L)	3.4 (1.5–5.5)	4.2 (1.1–12)	0.1617
Albumin (g/dL)	2.6 (2.3–2.8)	2.3 (2.1–2.5)	0.0001
Hematocrit (%)	41.2 (37–45.9)	35.1 (33–41.2)	0.0000
WBC (/mL)	13,045 (9680–17,930)	14,600 (9030–20,430)	0.5954
Neutrophils (%)	61 (47.3–72)	55 (40–75.1)	0.7930
NRBC (%)	0.4 (0–2.3)	1.6 (0–7.9)	0.1649
PLT (/mL)	229,500 (120,000–297,000)	57,000 (13,000–169,000)	0.0000
CRP (mg/L)	13.4 (3.4–40.2)	49.1 (7.2–85.2)	0.0010
SGOT (IU/L)	55 (33–83)	89 (54–235.5)	0.0000
SGPT (IU/L)	18 (12–30)	43 (23–141)	0.0000
T_BIL (mg/dL)	6 (3.8–9)	8.2 (3.9–25.4)	0.0053
D_BIL (mg/dL)	0.3 (0.2–0.4)	0.9 (0.3–17)	0.0000
BUN (mg/dL)	27.5 (19–47)	57 (35–92)	0.0000
Cr (mg/dL)	0.6 (0.4–0.8)	0.7 (0.5–1.2)	0.0105
Modified NEOMOD score	2 (1–4)	8 (5–10)	0.0000
SNAPPE score	9 (2–17)	19 (13–36)	0.0000
TOLLNER score	0.1 (0–3.5)	10 (0–13.5)	0.0019

Abbreviations: IQR: interquartile range; WBC, white blood count; NRBC, nucleated red blood cells; PLT, platelets; CRP, C-reactive protein; SGOT, serum glutamil oxaloacetic transaminase; SGPT, serum glutamic pyruvic transaminase; T_BIL, total bilirubin; D_BIL, direct bilirubin; BUN, blood urea nitrogen; Cr, creatinine plasma level; Modified NEOMOD score, modified Neonatal Multiple Organ Dysfunction scoring system; SNAPPE score, Score for Neonatal Acute Physiology with Perinatal extension.

**Table 4:** Univariate analysis results for morbidities affecting neonates’ survival.

Morbidity	OR and 95% CI	*p* Value
Acute Kidney Injury	28.65 (13.5–60.79)	<0.0001
Bronchopulmonary Dysplasia	0.3 (0.09–1)	0.0377
Disseminated Intravascular Coagulopathy	10.88 (5.6–21.15)	<0.0001
Intrauterine Growth Retardation	1.4 (0.59–3.31)	0.4388
Multi-organ Dysfunction Syndrome	32.7 (14.81–72.19)	<0.0001
Necrotizing Enterocolitis	2.82 (0.89–8.97)	0.0670
Patent Ductus Arteriosus	4.36 (1.7–11.16)	0.0009
Perinatal Hypoxia	2.27 (1.21–4.25)	0.0090
Periventricular Leucomalakia	14.52 (7.35–28.66)	<0.0001
Respiratory Distress Syndrome	5.61 (2.45–12.85)	<0.0001
Sepsis	2.58 (1.39–4.82)	0.0021

Abbreviations: OR, odds ratio; CI, confidence interval.

**Table 5:** Receiver Operating Characteristics curve analysis for NRBCs in study population.

	AUC	95% CI	*p* Value
Sepsis diagnosis	0.615	0.559–0.671	0.000
Hypoxia diagnosis	0.710	0.660–0.759	0.000
Mortality prognosis in all neonates	0.565	0.461–0.670	0.000
Mortality prognosis in septic neonates	0.760	0.631–0.888	0.001
Mortality prognosis in asphyxiated neonates	0.671	0.465–0.876	0.145

Abbreviations: AUC, area under the curve; CI, confidence interval.
